# Experimental Study on the Permeation and Migration Rules of Pressurized Water in Textile-Reinforced Concrete (TRC)

**DOI:** 10.3390/ma14216512

**Published:** 2021-10-29

**Authors:** Boxin Wang, Jiaqi Liu, Qing Wang

**Affiliations:** College of Construction Engineering, Jilin University, Changchun 130021, China; liujq19@mails.jlu.edu.cn (J.L.); wangqing@jlu.edu.cn (Q.W.)

**Keywords:** textile-reinforced concrete, durability, pressurized water, migration, permeability

## Abstract

As a new type of repairing and reinforcing material, textile-reinforced concrete (TRC) is often used to improve mechanical properties and durability of offshore, port, and hydraulic structures in the corrosive environment. In order to investigate how to quantify the permeability performance of TRC under external pressurized water, standard concrete permeability tests, nuclear magnetic resonance (NMR) tests, and scanning electron microscope (SEM) tests were conducted. These tests considered the effects of fiber grid size, Tex content, and water–cement ratio on the impermeability of TRC. Experimental results show that water gathers around the fiber bundles and migrates upwards along the longitudinal fiber under external water pressure and seeps out from the upper surface of the concrete specimen. Furthermore, based on the concentric annular slit flow theory and hydropower similarity principle, this study established a formula for the permeability of TRC and the calculated values are in good agreement with the experimental values.

## 1. Introduction 

The permeability of concrete to liquid and gas is an important physical property of concrete, which is closely related to the durability of concrete [1]. The permeability mainly depends on the pore structure [2,3] inside the matrix, and the durability of the structure repaired with textile-reinforced concrete (TRC) that can resist liquids and gases [4]. TRC is a new type of cement-based composite material consisting of multidimensional fiber bundles and concrete matrix [5,6]. Compared to reinforcement steel, textile fiber has the advantages of high corrosion resistance [7,8,9] and light weight [10]. Moreover, TRC can effectively resist the effects of chloride ions and carbon dioxide in the environment due to the presence of high corrosion resistance of the fiber materials [11] (such as carbon fiber, alkali-resistant glass fiber, basalt fiber).

TRC exhibits good potential to strengthen [12] and repair offshore structures and hydraulic structures in a harsh environment due to its good cracking resistance [13,14] and bearing capacity [15]. It can be used to limit various forms of concrete cracking, especially the cracking of mass concrete in concrete gravity dam, arch dam, and reinforced cement concrete gravity dam. TRC is also utilized for anti-cracking and anti-seepage of engineering structures, such as offshore platforms, harbor engineering, offshore large-volume concrete buildings, and hydraulic aqueducts. Therefore, studying the permeability of TRC for the repair and reinforcement of ocean engineering, port, and hydraulic structures is of great importance.

The permeability of concrete is related to the porosity [16] of the structure and external pressure [17]. In terms of the mechanical properties of TRC under complex environmental conditions, some scholars have done a lot of research and come to the conclusion that the presence of textile significantly improves the plasticity [18] and tensile strength [19] of concrete. Pourasee et al. [20] investigated the effect of fiber structure and yarn properties on the fluid transport properties of cracked cement composites. Yin et al. [21,22] explored the durability of concrete beams in continuous load salt attack environment by investigating the effect of TRC strengthening and salt corrosion use as a cross variable in the test. The results show that TRC has better ability to control crack development and good impermeability. Dolatabadi et al. [23] addressed a simple model to estimate quantity of penetration for fiber bundles based on cement particle size distribution and tow geometry. Victor Mechtcherine et al. [24,25,26] investigated the effects of multiple cracks on the permeability of water and gas in TRC and developed a model based on the Hagen–Poiseuille’s law to describe the effect of applied stress on the water permeability of cracked TRC. 

From the detailed review of existing literatures above, some investigations are found to be related with the permeability of TRC, but there is still a lack of study on the permeation and migration rules of pressurized water in TRC. Therefore, the objective of this study is to investigate the TRC permeability under pressurized water. Based on the concentric annular fracture flow theory and hydropower similarity principle, this study investigated the permeation and migration law of water in TRC under external hydraulic pressure. The accuracy of the theoretical model was verified by the concrete permeability tests. In addition, microscopic analysis of TRC permeability was performed by nuclear magnetic resonance (NMR) tests and scanning electron microscope (SEM) tests. The theoretical model can reflect the penetrations and migration of pressurized water in TRC with different fiber bundle spacings and textile contents under external hydraulic pressure.

## 2. Experiments and Results

### 2.1. Proportions of Concrete Mix

The concrete utilized in the test was cast using 42.5 R ordinary Portland cement, coarse aggregate with 5–10 mm, and fine river sand with a fineness modulus of 2.9. The two different mixture proportions of TRC used in this study are shown in Table 1.

### 2.2. Preparation of Textile

**Figure 1:**
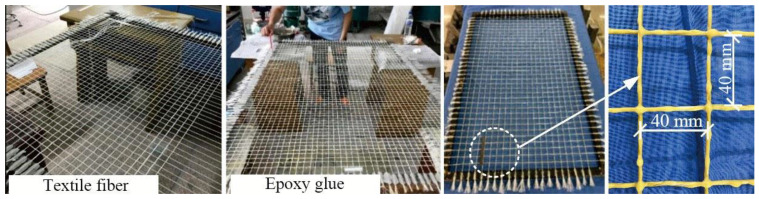
Textile.

Alkali-resistant glass fiber was selected with the knitting method of plain weave to make the textile, as shown in Figure 1. The glass fiber has low cost and its alkali resistance is greatly improved due to the existence of zirconia (ZrO_2_) [27,28,29]. Therefore, the glass fiber used in this study can effectively resist the corrosion of high alkaline substances in cement. The surface of fiber bundle was impregnated with epoxy resin adhesive, i.e., type CFSR-A/B impregnating adhesive produced by Carbon Technology Group Co., Ltd. The tensile strength of the epoxy resin is 57 MPa and the modulus of elasticity is 2.586GPa. The ratio of curing agent to diluent was set to 5:1 to improve the synergistic capacity of the fiber bundle and concrete matrix [30]. In the process of epoxy resin coating, the adhesive was impregnated between the roving inside the fiber bundle to completely bond the roving. The synergistic capacity of the fiber bundle and concrete matrix was improved after curing of the epoxy resin. The mechanical material performance of the glass fiber used in this study was tested in the Mechanics of Material Laboratory of Jilin University and the mechanical properties and geometric parameters of the alkali-resistant glass fiber are shown in Table 2 [31].

The cross-sectional shape of the fiber bundle is approximately circular because the textile was impregnated with the epoxy resin. The cross-sectional area of the fiber bundle was approximately calculated with the following formula and the equivalent radii of the fiber bundles with different Tex contents are shown in Table 3.

The Tex content is 9.2 k.

(1) $$A_{f}=\frac{m}{D_{f}}=\frac{2700\times 10^{-5}}{2.77}\times 10^{2}=0.975{\;\mathrm{mm}}^{2}$$

The Tex content is 18.4 k.

(2) $$A_{f}=\frac{m}{D_{f}}=\frac{2700\times 2\times 10^{-5}}{2.77}\times 10^{2}=1.95{\;\mathrm{mm}}^{2}$$

The Tex content is 27.6 k.

(3) $$A_{f}=\frac{m}{D_{f}}=\frac{2700\times 3\times 10^{-5}}{2.77}\times 10^{2}=2.925{\;\mathrm{mm}}^{2}$$

where $A_{f}$ is the cross-sectional area of the fiber bundle, mm^2^, m is the mass of 1000 m continuous fiber roving, g, and $D_{f}$ is the density of the fiber roving, g/cm^3^.

### 2.3. Specimen Grouping

The water–cement ratio of concrete, grid size, and Tex content of fiber bundles were taken as the test variables. The specific specimen parameters are shown in Table 4.

### 2.4. Specimen Preparation

**Figure 2:**
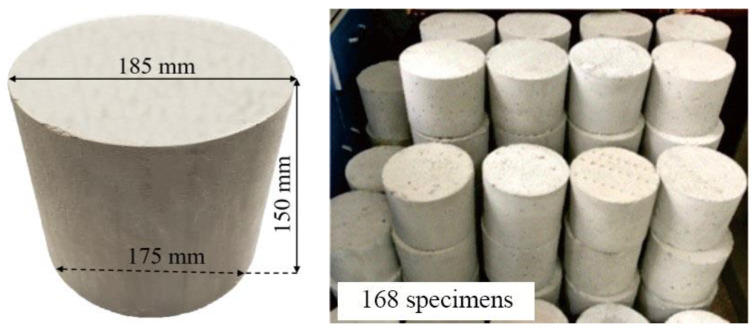
Detail of specimens.

The specimen was the frustum of a cone with an upper diameter of 175 mm, a lower diameter of 185 mm, and a height of 150 mm. A total of 168 specimens were used, as shown in Figure 2.

**Figure 3:**
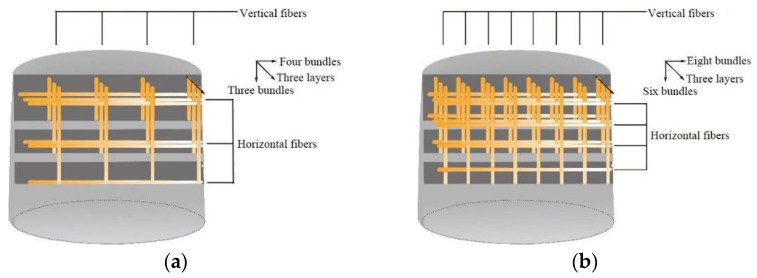
Fiber mesh layout; (**a**) Grid size 40 mm × 40 mm; (**b**) Grid size 20 mm × 20 mm.

Three layers of textile were placed parallel to the axis of the mold. The range of each layer consisted of 4 or 8 fiber bundles along the axial direction and 3 or 6 fiber bundles perpendicular to this direction, as shown in Figure 3.

### 2.5. TRC Permeability Test

**Figure 4:**
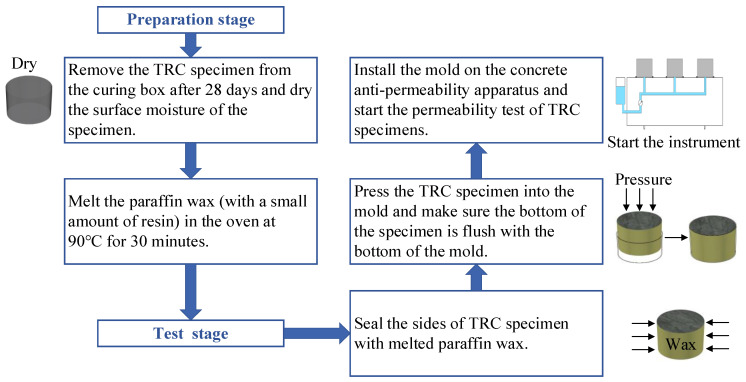
Flow chart of concrete permeability test.

**Figure 5:**
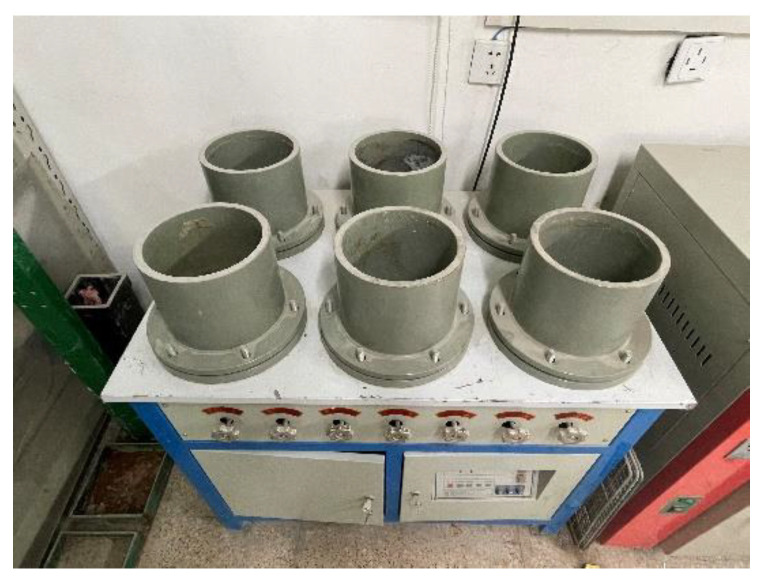
Concrete anti-permeability apparatus.

**Figure 6:**
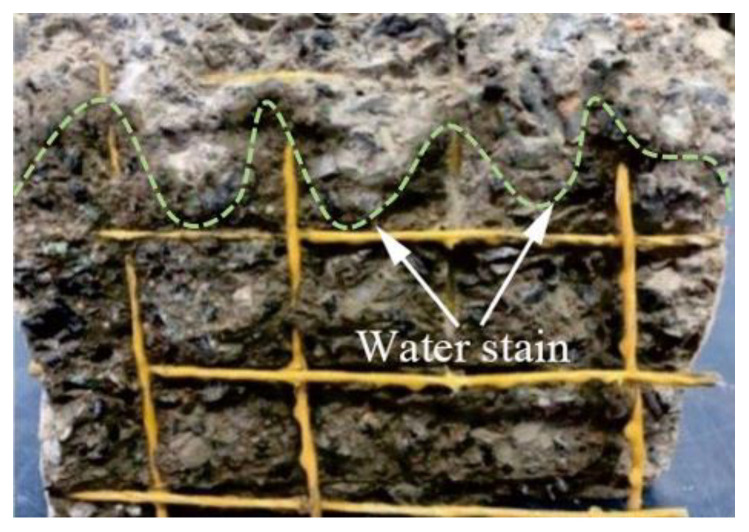
Water penetration path of the test piece.

The method of the TRC permeability test is shown in Figure 4 and the concrete anti-permeability apparatus is shown in Figure 5. The TRC specimens were 28 days of age and the water pressure of the permeability test was set to 0.5 MPa for 24 h. After the permeability test, the TRC specimens were split along the interface arranged by the textile in the TRC. Then, obvious water stains could be seen on the section, which represent the water seepage height, as shown in Figure 6. By comparing the water seepage height of each group of specimens, it was found that the water seepage height of specimens increased with the Tex content and water–cement ratio but decreased with the fiber grid size.

**Figure 7:**
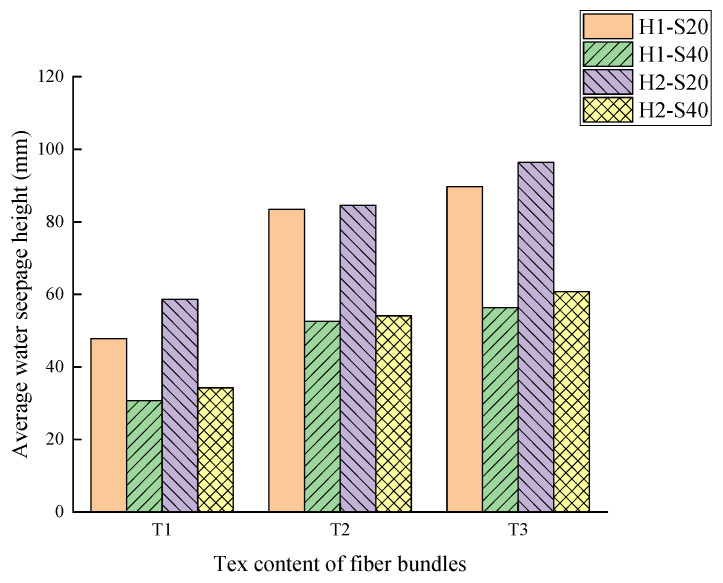
Average water seepage height.

The relative permeability coefficient and the average water seepage height of the TRC specimens under different research factors are summarized in Table 5 and Figure 7. According to the result of the TRC permeability test, the migration pattern of water in TRC can be described as the permeability of TRC increasing with the increase of Tex content and water–cement ratio but decreasing with the increase of fiber grid size.

### 2.6. Porosity Analysis

Nuclear magnetic resonance (NMR) relaxometry can carry out the detection of the porous structure in a completely nonperturbative manner [32,33]. Thus, NMR tests were conducted on the samples subjected to permeability tests for determining the pore characteristics of different fiber mesh spacings and different fiber contents in TRC. Before the NMR test, the specimens were placed in a vacuum saturation device for 24 h to make them reach the saturation state. Carr–Purcell–Meiboom–Gill pulse sequence was used to collect and calculate the NMR data. The calculation method is represented as Equation (4) [34].

(4) $$\frac{1}{T_{2}}\approx \frac{1}{T_{2\mathrm{surface}}}=\rho_{2}{\left(\frac{s}{v}\right)}_{\mathrm{hole}}$$

where $T_{2}$ is the transverse relaxation time of the pore fluid, ms; $T_{2\mathrm{surface}}$ is the transverse relaxation time caused by surface relaxation, ms; and $\rho_{2}$ is the surface relaxation intensity of $T_{2}$, μm/s. 

**Figure 8:**
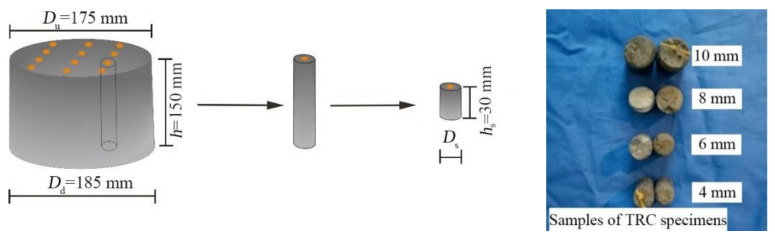
Sampling flow chart.

The specimens were carefully observed and sampled for NMR scanning after the permeability test. A circular truncated cone specimen was sampled randomly, as shown in Figure 8. The NMR samples were 4, 5, 6, 7, 8, 9, and 10 mm in diameter and 30 mm in height. To ensure the accuracy of the experimental data, there were three pieces of NMR samples of each size, and all samples were taken from the same TRC specimen. The pore parameters of the samples were measured and averaged with NMR apparatus (Suzhou Niumag Analytical Instrument Corporation, Suzhou, China) after sampling.

**Figure 9:**
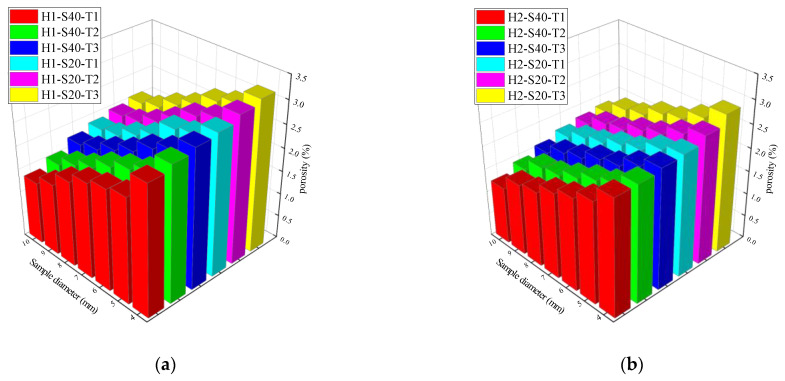
Porosities of TRC; (**a**) H1; (**b**) H2.

As shown in Figure 9, the porosity of the sample increased with the decrease in the radius of the specimen centered on the fiber bundle. The existence of the fiber bundle destroyed the integrity of the concrete matrix around it, resulting in its lowering. More pores were concentrated near the fiber bundles. Therefore, under the action of external water pressure, the free water migrated upward along the radial fiber bundle parallel to the external water pressure.

The NMR samples in different diameters all had large porosity and high permeability, indicating that there were many pores centered on fiber bundles inside the TRC specimen. As the water–cement ratio and the fiber grid size were constant, the porosity and permeability increased with the increase in the fiber bundle content. The diameter of the single-bonded fiber bundle increased, the specific surface area decreased, and the effective bond stress of the fiber bundle and the concrete matrix decreased with the increase in the fiber bundle content. Thus, the porosity and penetration rate increased. Combined with the results of the TRC permeability test and the NMR test, the rule can be described as the increase in the fiber content in the fiber bundle reduces the adhesion between the concrete matrix and the fiber bundle interface. Thus, the ratio of the gel pore to the capillary pore is reduced, and the proportion of noncapillary pores is increased. Therefore, increasing the fiber content in the fiber bundle increases the porosity and permeability. The performance results are consistent with the characteristics of the previous TRC permeability test.

NMR tests are performed through pulse sequence testing of hydrogenic ionization in water in a porous medium to obtain an attenuation signal. The attenuation amplitude can be fitted with a set of exponential decay curves, and the relaxation time is obtained by using a set of these decay constants. In porous media, the pore size is positively correlated with the relaxation time of H ions present in the pores. In accordance with the principle of NMR, the relationship between the relaxation time of water in the pore and the pore size can be expressed as

(5) $$\frac{1}{T_{2}}=\rho \frac{s}{V}$$

where ρ is the transverse surface relaxation strength of the porous medium, μm/ms, which is related to the sample types; s is the pore surface area; and V is the pore volume. The value of $T_{2}$ is related to the pore size and the distribution of $T_{2}$ spectrum indicates the distribution of the pores.

Assuming that the pore is an ideal sphere, the pore radius can be expressed as

(6) $$r=\mu T_{2}$$

where r is the pore radius and μ is the empirical transformation coefficient, which is related to the sample types.

The internal pore structure of the specimen changed with the addition of fiber. The value of $T_{2}$ can reflect the size of the pore diameter. Similarly, the distribution of the $T_{2}$ spectrum can represent the pore distribution. The amplitude is the signal intensity of $T_{2}$ spectrum. Thus, the higher the amplitude, the larger the porosity. 

**Figure 10:**
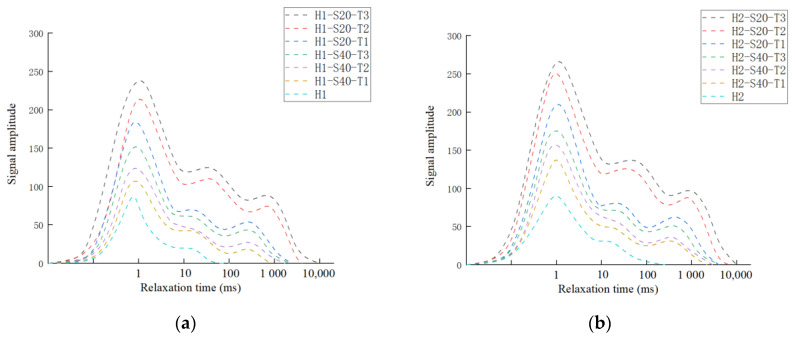
$T_{2}$ spectrum distribution curves; (**a**) H1; (**b**) H2.

The $T_{2}$ spectrum distribution curve is shown in Figure 10. The $T_{2}$ spectrum distribution curve of TRC contains three peaks, where the first peak corresponds to small pores, the second peak corresponds to medium pores, and the third peak corresponds to large pores and microcracks. When the grid size is constant, the $T_{2}$ spectrum curve shifts to the right with the increase in fiber bundle content. When the water–cement ratio is constant, the $T_{2}$ spectrum curve of the fiber mesh size of 20 mm × 20 mm shifts to the right compared with that of the fiber grid size of 40 mm × 40 mm. The increase in $T_{2}$ curve area indicates that the porosity of the sample increased. 

### 2.7. Microscopic Test Results

**Figure 11:**
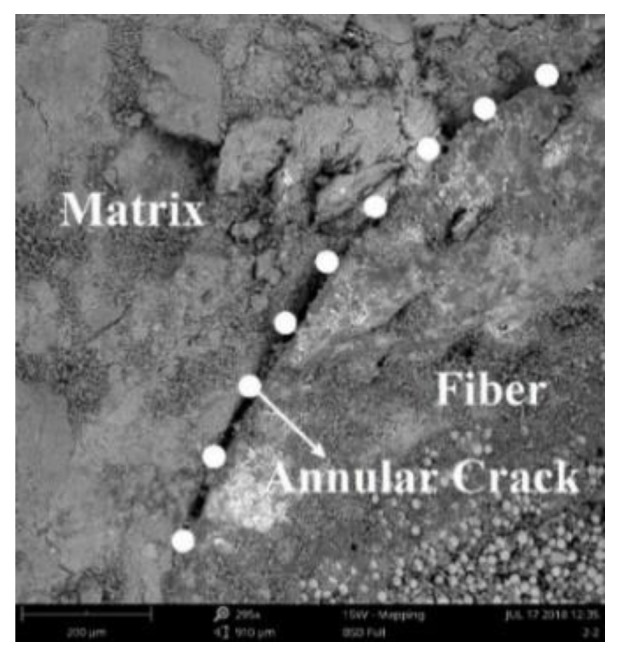
Annular crack in TRC.

Figure 11 shows a scanning electron microscopy (SEM) (Phenom-World, Eindhoven, Noord-Brabant, Netherlands) photograph of the fiber bundle and the concrete matrix in TRC after the permeability test. An annular crack was found between the fiber bundle and the concrete matrix. This phenomenon occurred because the bonding property between the oriented fiber bundle and the concrete wrapped by the epoxy resin binder was deficient, resulting in cracking between the interfaces.

**Figure 12:**
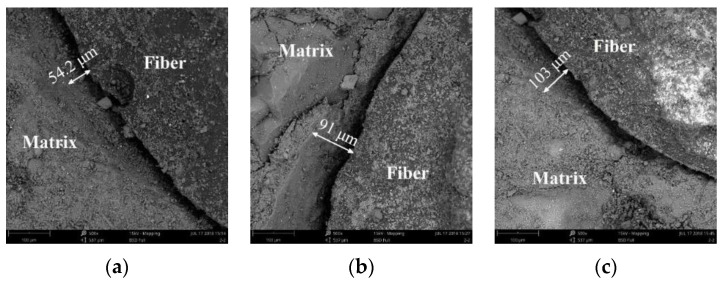
Annular crack width of specimens (water–cement ratio 0.45); (**a**) Tex content 9.2 k; (**b**) Tex content 18.4 k; and (**c**) Tex content 27.6 k.

**Figure 13:**
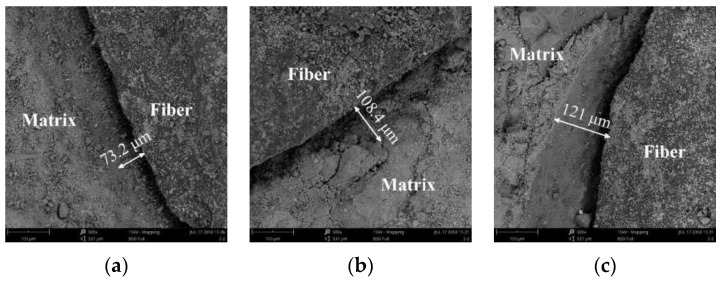
Annular crack width of specimens (water–cement ratio 0.55); (**a**) Tex content 9.2 k; (**b**) Tex content 18.4 k; and (**c**) Tex content 27.6 k.

Figure 12 and Figure 13 show the widths of annular crack between the concrete matrix and the fiber bundles in TRC with Tex content of 9.2 k, 18.4 k, and 27.6 k when the water–cement ratios were 0.45 and 0.55.

## 3. Theoretical Analysis

### 3.1. Mathematical Model of the Concentric Circular Slit Flow

**Figure 14:**
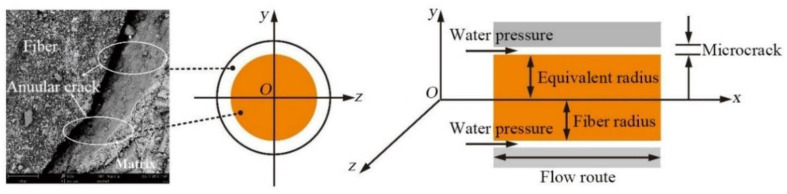
Concentric annular slit.

In the TRC, microcracks are found on the interface between the fiber bundles and the concrete matrix. Therefore, the pressurized water migrated in the microcracks along the fiber bundle. The microcracks around this fiber bundle can be expressed as the concentric circular slit, as shown in Figure 14.

The migration and flow of pressurized water in the concentric annular slit was described with the Navier–Stokes equation [35], which is a general equation describing the motion of viscous fluids. In the slit flow, the viscous force is dominant, whereas the mass force is negligible. The mathematical model of the concentric ring slit flow under the dynamic boundary condition is simplified based on the following assumptions.

(1) The water migrates at a constant velocity in the cylindrical pipe.

(2) The cylinder is extremely long to ignore the influence of the end face.

(3) The flow of water is steady. 

Therefore, the governing equation of the concentric ring slit flow under dynamic boundary condition (only considering axial motion) can be expressed as

(7) $$f_{x}-\frac{1}{\rho }\frac{\partial P}{\partial x}+v\left(\frac{\partial^{2}u_{x}}{\partial x^{2}}+\frac{\partial^{2}u_{x}}{\partial y^{2}}+\frac{\partial^{2}u_{x}}{\partial z^{2}}\right)=\frac{\partial u_{x}}{\partial t}+u_{x}\frac{\partial u_{x}}{\partial x}+u_{y}\frac{\partial u_{x}}{\partial y}+u_{z}\frac{\partial u_{x}}{\partial z}\;$$

where $f_{x}$ is the mass force; P is the dynamic water pressure; $u_{x}$ is the rate of axial slit flow; ρ is the fluid density; v is the kinematic viscosity.

The crevice flow under dynamic boundary condition only considers the axial movement of the pipe and is a constant flow, and the mass force is only gravity. Thus, the following conditions can be obtained.

(8) $$u_{y}=u_{y}=0,\;\frac{\partial u_{x}}{\partial t}=0,\;f_{x}=0$$

(9) $$u_{y}\left(\frac{\partial u_{x}}{\partial y}\right)=0,\;u_{z}\left(\frac{\partial u_{z}}{\partial z}\right)=0,\;\frac{\partial u_{y}}{\partial y}=0,\;\frac{\partial u_{z}}{\partial z}=0$$

(10) $$\frac{\partial u_{x}}{\partial x}+\frac{\partial u_{y}}{\partial y}+\frac{\partial u_{z}}{\partial z}=0$$

(11) $$\frac{\partial u_{x}}{\partial x}=0,u_{x}\left(\frac{\partial u_{x}}{\partial x}\right)=0,\;\frac{\partial^{2}u_{x}}{\partial x^{2}}=0$$

Therefore, Equation (7) can be simplified as follows:

(12) $$\frac{\partial P}{\partial x}=\rho \nu \left(\frac{\partial^{2}u_{x}}{\partial y^{2}}+\frac{\partial^{2}u_{x}}{\partial z^{2}}\right)$$

$u_{x}$ does not change along x because $\frac{\partial u_{x}}{\partial x}=0$. From Equation (12), $\frac{\partial P}{\partial x}$ is independent of x. Thus, $\frac{\partial P}{\partial x}$ is a constant that can be expressed as

(13) $$\frac{\partial P}{\partial x}=-\frac{∆P}{L}$$

The concentric annular crack flow is axisymmetric, $\frac{\partial^{2}u_{x}}{\partial y^{2}}$ is the same as $\frac{\partial^{2}u_{x}}{\partial z^{2}}$, and y and z are along the radial direction. Thus, variables y and z can be replaced with variables r, $u_{x}$, and x. Variable $u_{x}$ is related to r only.

(14) $$\frac{\partial^{2}u_{x}}{\partial y^{2}}=\frac{\partial^{2}u_{x}}{\partial z^{2}}=\frac{d^{2}u_{x}}{dr^{2}}$$

Substitute Equation (14) into Equation (12) and integrate it.

(15) $$u_{x}=-\frac{\Delta P}{4L\mu }r^{2}+C_{1}r+C_{2}$$

In accordance with the boundary conditions, when r=R, ux=0; when $r=r_{0}$, $u_{x}=0$, and substitute into Equation (15)

(16) $$C_{1}=\frac{\Delta P}{4L\mu }\left(R+r_{0}\right)$$

(17) $$C_{2}=-\frac{\Delta P}{4L\mu }Rr_{0}$$

Substituting $C_{1}$ and  into Equation (15) obtains the flow velocity at any point along the radius.

(18) $$u_{x}=-\frac{\Delta P}{4L\mu }r^{2}+\frac{\Delta P}{4L\mu }\left(R+r_{0}\right)r-\frac{\Delta P}{4L\mu }Rr_{0},\;r_{0}<r<R$$

Therefore, the total flow through the annular crack section is

(19) $$Q=\int_{r_{0}}^{R}u_{x}2\pi \left(r-r_{0}\right)dr=-\frac{\pi ∆P}{24L\mu }\left(7R^{4}-12R^{3}r_{0}+6R^{2}r_{0}{}^{2}+3r_{0}{}^{4}-4Rr_{0}{}^{3}\right)$$

The average flow velocity of the annular crack section is

(20) $$\upsilon =\frac{Q}{\pi \left(R^{2}-r_{0}^{2}\right)}=-\frac{∆P\left(7R^{4}-12R^{3}r_{0}+6R^{2}r_{0}^{2}+3r_{0}^{4}-4Rr_{0}^{3}\right)}{24L\mu \left(R^{2}-r_{0}^{2}\right)}$$

### 3.2. TRC Penetration Model

#### 3.2.1. Introduction of the Model

In the actual engineering, TRC is often used in complex environments that are affected by many types of water migration and fissure seepage. The change in permeability is closely related to the development degree and tissue morphology of the fracture system [36].

**Figure 15:**
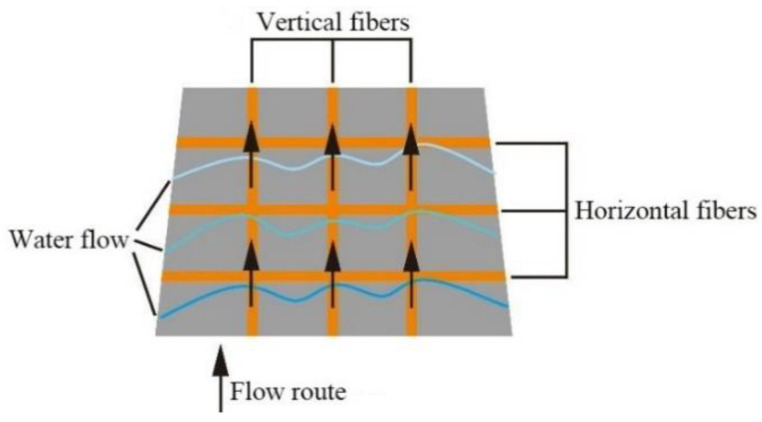
Bidirectional joint cracks.

An equivalent seepage resistance method was adopted to study the migration law of water pressure in TRC. Specifically, the hydroelectric similarity principle was used to describe the seepage field with circuit diagram, and then the circuit law was applied to solve the seepage law. In TRC masses, the main fracture system was composed of a finite number of single fractures. Only two basic combinations (series and parallel) were found between each single crack. The layout of the fiber in the TRC created a mutually perpendicular joint crack in TRC (Figure 15). Thus, the main factor of the permeability of the TRC depends on the joint cracks.

#### 3.2.2. Model Establishment

Several fundamental assumptions were suggested for the penetration model.

**Figure 16:**
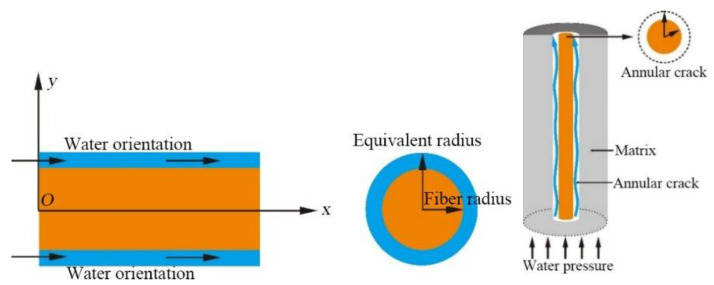
Concentric annular crack of fiber bundle.

(1) The self-healing effect of concrete cracks is disregarded.

(2) During the application of water pressure, a small amount of water permeates along the transverse fibers, which is negligible.

(3) The fiber bundle is a homogeneous isotropic linear elastic cylinder.

(4) A concentric annular crack is found on the fiber bundle at the interface between the fiber and the concrete, as shown in Figure 16.

In accordance with the above fundamental assumptions, the longitudinal fiber bundles are responsible for the permeability of the TRC under the external pressure water environment. Only the parallel gap between the longitudinal fiber bundles and the concrete matrix was considered.

**Figure 17:**
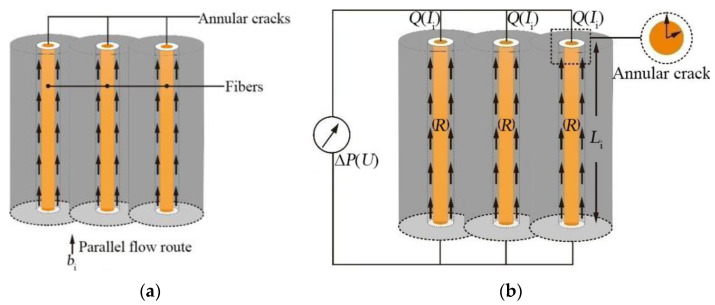
Schematic diagram of hydropower similarity principle; (**a**) parallel cracks; (**b**) analog circuit.

As shown in Figure 17a, cracks were connection in parallel. Therefore, the total flow in the crack system was equal to the sum of the flow in each crack, which can be obtained as Equation (21).

(21) $$Q_{f}=Q_{f1}+Q_{f2}+Q_{f3}$$

Substitute Equation (19) into Equation (21) to obtain

(22) $$Q_{f}=Q_{f1}+Q_{f2}+Q_{f3}=\sum_{i=1}^{3}\frac{\Delta P}{\frac{L_{i}}{\frac{\pi }{24\mu }\left(7R_{i}^{4}-12R_{i}^{3}r_{i}+6R_{i}^{2}r_{i}^{2}+3r_{i}^{4}-4R_{i}r_{i}^{3}\right)}}$$

In the circuit analysis, the formula of the parallel circuit is $I=\frac{U}{I_{1}}+\frac{U}{I_{2}}$. In accordance with Equation (19), the parallel circuit relationship in the crack seepage can be defined as follows:

(23) $$R=\frac{L_{i}}{\frac{\pi }{24\mu }\left(7R_{i}{}^{4}-12R_{i}{}^{3}r_{i}+6R_{i}{}^{2}r_{i}{}^{2}+3r_{i}{}^{4}-4R_{i}r_{i}{}^{3}\right)}$$

Equation (23) plays an important role in the equation, which is similar to resistance R in the circuit. R is referred as the equivalent seepage resistance of the fracture.

The fluid seepage in the regional trunk crack can be equivalent to the parallel circuit group in the analog circuit, and the similarity between them is given in Figure 17b.

### 3.3. TRC Penetration Model

Assuming that a regional backbone (the longitudinal fibers in TRC) has several parallel equivalent seepage resistances, the equivalent seepage resistance Rf in the main fracture system of this region is

(24) $$R_{f}=\sum_{j}[\frac{L_{i}}{\frac{\pi }{24\mu }\left(7R_{i}{}^{4}-12R_{i}{}^{3}r_{i}+6R_{i}{}^{2}r_{i}{}^{2}+3r_{i}{}^{4}-4R_{i}r_{i}{}^{3}\right)}]$$

Thus, the TRC system flow is

(25) $$q=\frac{∆P}{R_{f}}=\frac{∆P}{\sum_{j}[\frac{L_{i}}{\frac{\pi }{24\mu }\left(7R_{i}{}^{4}-12R_{i}{}^{3}r_{i}+6R_{i}{}^{2}r_{i}{}^{2}+3r_{i}{}^{4}-4R_{i}r_{i}{}^{3}\right)}]}$$

The relationship between the permeability k of the fracture system and the equivalent seepage resistance is

(26) $$k=\frac{q\mu L}{A\Delta P}=\frac{\mu L}{AR_{f}}$$

Therefore, the penetration rate of the TRC system is

(27) $$k=\frac{q\mu L}{A\Delta P}=\frac{\mu L}{AR_{f}}=\frac{∆x}{A\sum_{j}[\frac{L_{i}}{\frac{\pi }{24\mu }\left(7R_{i}{}^{4}-12R_{i}{}^{3}r_{i}+6R_{i}{}^{2}r_{i}{}^{2}+3r_{i}{}^{4}-4R_{i}r_{i}{}^{3}\right)}]}$$

**Figure 18:**
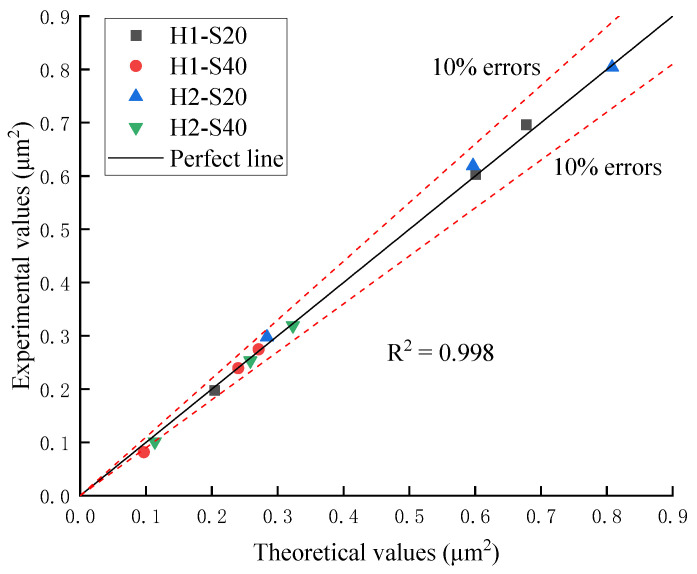
Comparison between the experimental and theoretical values.

The contrastive between the experimental and theoretical values of TRC permeability are shown in Table 6 and Figure 18.

## 4. Discussion

**Figure 19:**
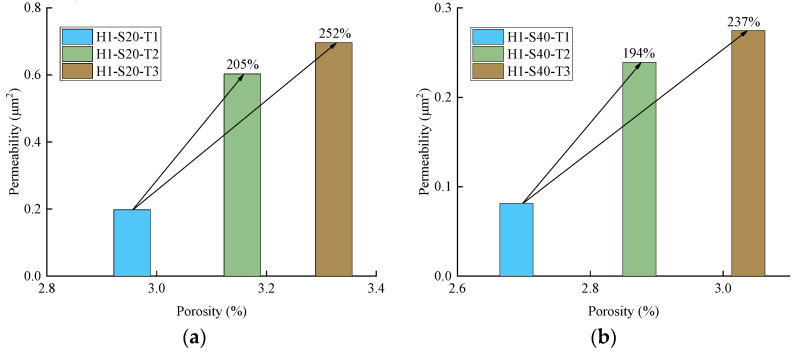
Relationship between the permeability and porosity of TRC (H1); (**a**) Grid size 20 mm × 20 mm; (**b**) Grid size 40 mm × 40 mm.

**Figure 20:**
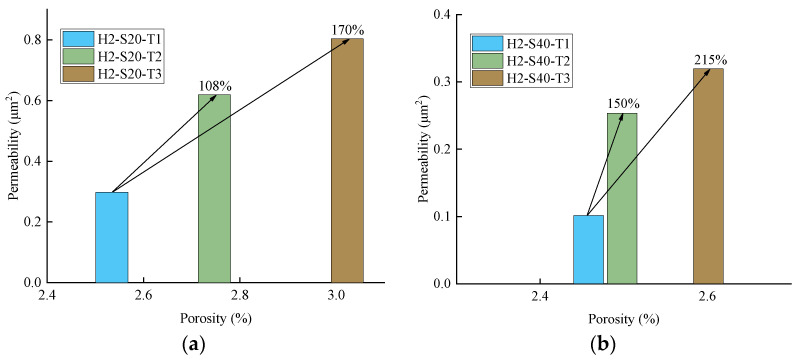
Relationship between the permeability and porosity of TRC (H2); (**a**) Grid size 20 mm × 20 mm; (**b**) Grid size 40 mm × 40 mm.

In order to explore the permeation and migration law of pressurized water in TRC, a permeability test of TRC was conducted in this study. According to the results of the permeability test, it can be concluded that the permeability of TRC increases with increasing Tex content and water–cement ratio but decreases with increasing grid size. For the purpose of investigating the microscopic mechanism leading to the above phenomenon, NMR tests and SEM tests were conducted on the specimens that had undergone the permeability test. The microscopic test results corroborated the results of the permeability test well, the porosity increased with increasing Tex content and water–cement ratio but decreased with increasing grid size. The relationship between the permeability of TRC and the porosity of the NMR sample with a diameter in 4 mm is shown in Figure 19 and Figure 20. Therefore, the law that the permeability of TRC increases with porosity can be found intuitively.

In addition, an annular crack was found between the fiber bundle and the concrete matrix by the SEM tests. These test results above indicate that due to the presence of fiber bundles, pores and microcracks were found in the concrete matrix near the fiber bundles, so water certainly permeated and migrated along the fiber bundle in TRC under the action of external pressure. In terms of theoretical analysis, based on the concentric annular fracture flow theory and hydropower similarity principle, the permeation and migration rules of pressurized water in TRC were investigated. The accuracy of the theoretical model was verified by the concrete permeability tests and the theoretical model can reflect the permeation and migration of pressurized water in TRC with different grid sizes and Tex contents.

## 5. Conclusions

In this study, the permeation and migration rules of pressurized water in TRC were investigated through theoretical and experimental approaches. The conclusions are summarized as follows:

(1) The water penetrated and migrated along the direction of water pressure around the longitudinal fiber bundle inside the TRC under the effect of external water pressure.

(2) Annular cracks were found around the fiber bundle and the concrete matrix through the SEM test, and the microcracks between the fiber bundle and the concrete matrix were measured by IPP.

(3) The penetration and migration rules of pressurized water in TRC and the calculation formula of its permeability were established based on concentric annular fracture flow theory and hydropower similarity principle. The theoretical model can reflect the penetration and migration of pressurized water in TRC with different fiber bundle spacings and fiber contents well. The calculated values of TRC permeability were in good agreement with the experimental values, and the errors between them were less than 18.8%.

## Figures and Tables

**Table 1:** Mixture proportions of concrete (kg/m^3^).

Water Cement Ratio	Water	Cement	Sand	Stone
0.45	185	411	545	1158
0.55	185	337	633	1125

**Table 2:** Material properties of the alkali-resistant glass fiber.

Tensile Strength (MPa)	Elastic Modulus (GPa)	Density (g/m^3^)	Sectional Area (mm^2^)
1600	72	2700	0.975

**Table 3:** Equivalent radius of fiber bundles with different Tex contents.

Tex Content	9.2 k	18.4 k	27.6 k
Equivalent radius	0.557 mm	0.788 mm	0.965 mm

**Table 4:** Differences of specimens.

Specimen Number	Water-Cement Ratio	Grid Size	Tex Content	Number
H1-S20-T1	0.45	20 mm × 20 mm	9.2 k × 9.2 k	12
H1-S20-T2	0.45	20 mm × 20 mm	18.4 k × 18.4 k	12
H1-S20-T3	0.45	20 mm × 20 mm	27.6 k × 27.6 k	12
H1-S40-T1	0.45	40 mm × 40 mm	9.2 k × 9.2 k	12
H1-S40-T2	0.45	40 mm × 40 mm	18.4 k × 18.4 k	12
H1-S40-T3	0.45	40 mm × 40 mm	27.6 k × 27.6 k	12
H2-S20-T1	0.55	20 mm × 20 mm	9.2 k × 9.2 k	12
H2-S20-T2	0.55	20 mm × 20 mm	18.4 k × 18.4 k	12
H2-S20-T3	0.55	20 mm × 20 mm	27.6 k × 27.6 k	12
H2-S40-T1	0.55	40 mm × 40 mm	9.2 k × 9.2 k	12
H2-S40-T2	0.55	40 mm × 40 mm	18.4 k × 18.4 k	12
H2-S40-T3	0.55	40 mm × 40 mm	27.6 k × 27.6 k	12

**Table 5:** Permeability test results.

Specimen Number	Average Water Seepage Height (mm)	Relative Permeability Coefficient (mm/h)
H1-S2-T1	30.67	7.35 × 10^−6^
H1-S2-T2	52.56	2.16 × 10^−5^
H1-S2-T3	56.32	2.48 × 10^−5^
H1-S4-T1	47.78	1.78 × 10^−5^
H1-S4-T2	83.45	5.44 × 10^−5^
H1-S4-T3	89.69	6.28 × 10^−5^
H2-S2-T1	34.23	9.15 × 10^−6^
H2-S2-T2	54.11	2.29 × 10^−5^
H2-S2-T3	60.73	2.88 × 10^−5^
H2-S4-T1	58.62	2.68 × 10^−5^
H2-S4-T2	84.57	5.59 × 10^−5^
H2-S4-T3	96.37	7.26 × 10^−5^

**Table 6:** Comparison between the experimental and theoretical values.

Specimen Number	Experimental Values (μm^2^)	Theoretical Values (μm^2^)	Errors (%)
H1-S20-T1	0.19762	0.20428	3.370
H1-S20-T2	0.60281	0.60031	0.415
H1-S20-T3	0.69633	0.67764	2.684
H1-S40-T1	0.08142	0.0967	18.767
H1-S40-T2	0.23913	0.24012	0.414
H1-S40-T3	0.27457	0.27106	1.278
H2-S20-T1	0.29746	0.28374	4.612
H2-S20-T2	0.6191	0.59667	3.623
H2-S20-T3	0.80392	0.80789	0.494
H2-S40-T1	0.10142	0.11338	11.793
H2-S40-T2	0.25345	0.25867	2.060
H2-S40-T3	0.31925	0.32316	1.225

## Data Availability

The data presented in this study are available on request from the corresponding author. The data are not publicly available due to [the data also forms part of an ongoing study].
